# Shopping versus Nature? An Exploratory Study of Everyday Experiences

**DOI:** 10.3389/fpsyg.2018.00009

**Published:** 2018-01-23

**Authors:** Tony P. Craig, Anke Fischer, Altea Lorenzo-Arribas

**Affiliations:** ^1^Social, Economic and Geographical Sciences Research Group, The James Hutton Institute, Aberdeen, United Kingdom; ^2^Biomathematics and Statistics Scotland, Aberdeen, United Kingdom

**Keywords:** nature, shopping, experience, connectedness to nature, emotions

## Abstract

Although a growing volume of empirical research shows that being in nature is important for human wellbeing, the definition of what constitutes an ‘experience in nature,’ and how this is different from other types of experiences, is very often left implied. In this paper we contrast everyday experiences involving nature with a category of everyday experience in which most people regularly partake. We present an exploratory study in which people (*N* = 357) were explicitly asked to describe a memory they had of an everyday ‘experience which involved nature,’ as well as an everyday ‘experience which involved shopping.’ The open-ended responses to these questions were analyzed both quantitatively and qualitatively. Nature experiences were generally found to be more positive than shopping experiences, and they were more likely to be rated as ‘peaceful’ and ‘active’ compared to shopping experiences. Follow-up analyses indicate a significant interaction between experience category (nature or shopping), and the relationship between connectedness to nature and the amount of pleasure associated with that experience: The more strongly connected to nature a respondent was, the larger the disparity between the pleasantness of the shopping experience and that of the experience in nature tended to be.

## Introduction

There is a growing volume of empirical research demonstrating that being in nature is important for human wellbeing, as it, for example, facilitates attention restoration (Kaplan, 1995; Hartig et al., 1996; Berto, 2005) and has positive impacts on health (de Vries et al., 2003). In line with this, visualizations of natural environments are usually perceived as being more beautiful (Van der Jagt et al., 2014) and to possess a higher number of restorative characteristics than built ones (Laumann et al., 2001). Findings such as these have often been regarded as support for the assumption of a ‘biophilia,’ i.e., an (innate) love for nature (Van den Born et al., 2001). At the same time, however, being in nature can also evoke stress and feelings of fear (Bixler and Floyd, 1997; Van den Berg and ter Heijne, 2005).

Research on these effects has referred to a number of different representations of nature, ranging from static representations such as pictures (Van Den Berg and Koole, 2006; Van der Jagt et al., 2014) to imagined scenarios (Van den Berg and ter Heijne, 2005) and real environments (Tyrväinen et al., 2014), and from parks (Tyrväinen et al., 2014) to forests (Dandy and Van Der Wal, 2011) and wilderness areas (Van Den Berg and Koole, 2006). However, while the specific examples used to represent nature in these studies are usually very clear and concrete (for example, depicted in a photograph), a large number of studies draw on researcher-defined concepts of nature that arguably do not account for idiosyncratic interpretations by the individual, but tend to assume that on average such variability in interpretation is not problematic at such higher levels of abstraction. This holds similarly for studies that conceptualize nature in abstract, theoretical terms, such as the work on connectedness to nature (Mayer and Frantz, 2004). Apart from a small number of studies concerning concepts of nature as such (Buijs, 2009; Buijs et al., 2009), perceptions of naturalness of foods (Rozin, 2006) or materials like wood or stone (Overvliet and Soto-Faraco, 2011), many studies in this research domain take ‘naturalness’ as a pre-defined category. We intend to take a step back from any pre-defined conceptualizations, and ask what an “experience involving nature” actually means, investigating in a grounded manner how individuals (as opposed to researchers) conceptualize everyday experiences with the category “nature.” Ives et al. (2017) note that literature on the human-nature connection (HNC) can be broadly categorized as focusing on HNC as mind, HNC as place, and HNC as experience. Our research clearly falls into the “HNC as experience” category.

We conceptualize everyday experiences here as a person-environment relationship using the perspective of the “lived day of an individual” (Craik, 2000). In broad terms, our focus is on how episodes of an individual’s day can be categorized, and how certain categories of experience compare to each other from the perspective of an individual. Whilst some categories of activity are likely to be associated with a relatively shared understanding of what that activity involves (e.g., sleeping), other categories of activity (e.g., leisure, childcare, or shopping) are less agreed-upon as categories of experience. In many ways this approach is similar to that followed by sociological studies of time use (e.g., Gershuny, 1987; Sullivan, 2008), insofar as it is accepted that everyday life is usefully understood through the categorization of activities and experiences as they are arranged across a 24-h cycle. In our study, we examine how the everyday experiences associated with the category ‘nature’ are perceived and evaluated. In contrast to the majority of other studies looking at nature experiences that, at best, compare (for example) a walk through a forest to a walk through an urban center (Tyrväinen et al., 2014), we explore here comparisons with a different everyday experience, in this case shopping. Although previous research studies have looked at aspects of the shopping experience in more detail than we have, including measures such as shopping enjoyment scales (Roehm et al., 2002) and scales designed to measure the attractiveness of shopping environments (Hart et al., 2007), we are not aware of any studies that have directly compared shopping and nature experiences.

It has been argued that in many contemporary societies, the role of shopping in people’s lives goes far beyond the provision of food and other necessary household items and has important symbolic and recreational functions such as those associated with identity and status (Falk and Campbell, 1997; Miller, 1998; Shaw, 2010). And even though sociological analyses of shopping (just like psychological studies of nature experiences) rarely discuss these functions in relation to those of other activities, we suggest here that in terms of specific experiences within the superordinate category, shopping might sometimes complement or even displace other symbolic and recreational experiences, such as those within the superordinate category of nature-related experiences. For example, the very existence of terms such as ‘retail therapy’ – even if these are usually employed tongue-in-cheek – suggests that shopping is by many people seen as an experience that can alleviate distress and sadness (Atalay and Meloy, 2011; Rick et al., 2014). Gilboa and Vilnai-Yavetz (2010) describe how use and enjoyment of shopping in malls varies between age-groups. Conversely, nature-related experiences are by no means seen as desirable or attractive by all citizens. Where these issues are discussed, the focus has been on the role of sociological constructs like social class and ethnicity (Suckall et al., 2009). A study of school children in Northern England (Suckall et al., 2009) found that moorland landscapes tended to be preferred by middle-class children, and that shopping environments were more likely to be favored by working-class children. However, such studies are still largely based on reactions to predefined stimuli or descriptions rather than to personally relevant experiences. Notable exceptions might be the explorations of Jones (1999) and Arnold et al. (2005) using the Critical Incident technique that allowed the identification of factors that made shopping either pleasurable and fun, or unpleasant and unentertaining. Kloek et al. (2015) found in their culturally diverse Dutch sample that 40% of all non-immigrant respondents and 58% of all Chinese immigrant respondents had not engaged in nature-based recreation in the widest sense in the last 3 months – for a large part of these, a lack of interest (rather than other constraints such as time or accessibility) was the main reason. Interestingly, such studies, which explicitly take into account the possibility that experiences can be unpleasant and elicit the factors that constitute negative experiences, seem to be largely missing (with some notable exceptions such as Bixler and Floyd, 1997; Van den Berg and ter Heijne, 2005) from the literature on human-nature relationships. Here, we do not make any *a priori* assumptions about the valence of an experience, but contrast two types of experiences that, in principle, could be both either recreational or part of an everyday chore (e.g., in the case of shopping, the regular purchase of food or cleaning products, or in the case of nature, the clearing of snow from a pavement, or lawn mowing), and that could both be perceived as either pleasant or unpleasant (or, in fact, neutral). We investigate neither practices nor social representations (Buijs et al., 2012) related to nature or shopping. Rather, we explore how people recall their own experiences with nature and shopping, and how they describe and characterize these. We then investigate how these perceptions and evaluations sit alongside and relate to other relevant cognitions.

In our examination of how people perceive and evaluate everyday experiences, we assume that experiences with nature are not homogeneously positive, and explore potential explanations for such variation. Connectedness with nature has been put forward as a stable construct that captures an individual’s emotional relationship with nature (Mayer and Frantz, 2004; Mayer et al., 2009), and this construct may account for some variation in the way people evaluate nature experiences, for example, in terms of the well-being derived from these experiences. Many studies exploring the relationship between exposure to nature and wellbeing have shown that spending time in, or living near to, natural environments tends to be associated with increased well-being compared to other environments (Kaplan, 2001; Maas et al., 2009). That is not to suggest that human-made environments are always somehow ‘worse’ than natural environments when it comes to well-being. Rather, one might say that there appears to be a higher likelihood of the presence of properties related to psychological well-being in environments that are categorized as being ‘natural.’ In addition, Mayer and Frantz (2004) suggest that measures of connectedness with nature (as a stable trait) should be positively associated with measures of subjective well-being.

In a recent study exploring this relationship in further detail, Zhang et al. (2014) found that emotional engagement with nature moderated the relationship between connectedness with nature and psychological well-being. While this essentially shows that closeness to nature and wellbeing are only connected for those individuals who experience nature as emotionally positive, we move the debate here a step further by asking if connectedness to nature interacts with the evaluation of everyday experiences with nature or shopping. In terms of nature experiences, we would expect that people with high levels of nature connectedness would find ‘nature experiences’ more pleasurable, and on average more positive than people with lower levels of connectedness. Conversely, and following a similar logic, we also aim to explore if people high in ‘nature connectedness’ evaluate shopping experiences more negatively than people scoring lower on measures of connectedness to nature. We are thus allowing for the possibility that nature-based experiences are not homogeneously positive, as previous literature has suggested that quite simply people sometimes prefer not to engage in nature-related activities.

Here, we explore how people recall everyday experiences linked to two broad category-words – namely those associated with the terms ‘nature’ and ‘shopping.’ Whilst there has been considerable research in both of these domains separately, to our knowledge this is the first study to compare nature and shopping experiences within the same study. We were interested to know what people understood ‘an experience involving nature’ to mean, and how such experiences were described in comparison to shopping experiences. In addition, we wanted to better understand how people evaluated these nature experiences relative to shopping experiences, to investigate the role of connectedness to nature as a stable trait in moderating these evaluations.

## Materials and Methods

### Participants

A convenience sample was recruited consisting of 357 participants (57% female). The mean age of participants was 39 (*SD* = 13.8). Thirty-five percent of all participants had children, and 57% had completed a university-level qualification. The majority, namely 271 of these participants, were recruited via an on-line survey described as an “everyday experiences questionnaire,” and the remaining 86 participants were approached in public places in the city center of Aberdeen, United Kingdom, and asked to fill in a paper-version of the questionnaire. The reason for adding the face-to-face sample was to maximize the variability of responses to the shopping question (because people approached in the city center were very likely to have been shopping recently). Exploratory analysis of the differences between the two samples found that as expected, the face-to-face sample tended to rate the shopping experience as more pleasant and peaceful when compared with the online sample. Given the exploratory nature of this study, we therefore decided that the additional variation in the range of responses added by the face-to-face sample justified the data being included in the overall data set. Due to anonymity and the convenience nature of the sample, we do not have sufficient information about the respondent’s place of residence. However, from the descriptions of experiences, we can infer that the majority were based in the north east of Scotland.

### Measures

#### Recall of Everyday Experiences

We used an autobiographical memory retrieval method similar to that described by Kopelman et al. (1989), where participants are required to recall autobiographical memories in response to a cue word or phrase. Participants were told that the questionnaire was about “everyday experiences” and were then asked two open ended questions of the form: “*Please describe a recent experience you have had which you would describe as:”* followed by (1) “*An experience which involved nature*” and (2) “*An experience which involved shopping*.” The order of question presentation was not counterbalanced, so participants always answered the question about nature first. Participants were asked to write a few sentences describing the experience that came to mind. Following this, participants were asked to rate their self-elicited experience on eight 7-point semantic differential scales: ‘*familiar – unfamiliar*’, ‘*pleasant – unpleasant*,’ ‘*active – passive*,’ ‘*arousing – not arousing*,’ ‘*simple – complex*,’ ‘*peaceful – not peaceful*,’ ‘*in control – not in control*,’ ‘*difficult – easy*.’

#### Nature Connectedness

Connectedness to nature was measured using the ‘connectedness to nature scale’ (Mayer and Frantz, 2004), which asks people to express level of agreement with 14 items related to the level of (dis)connection with nature. The scale has been used in many previous studies (Capaldi et al., 2014), and has been shown to have robust psychometric properties, which is why we selected it for the current study. For the statistical analysis, when analyzing the connectedness to nature scale, one item (“*I often feel a kinship with animals and plants*”) was not included in the paper version of the questionnaire by mistake; therefore, all analyses were done on a 13-item version of the scale, with a Cronbach’s alpha of 0.81.

### Procedure

For both the paper-and-pencil and the on-line version of the questionnaire, participants were provided with a page of information about the study and the wider project of which this study was part, and then given assurances of anonymity, before asking for written confirmation of informed consent. The only notable difference between the on-line and the paper-and-pencil version of the questionnaire was that we added some data-checks to the on-line version to minimize missing data. The question order was the same for both the paper and the on-line questionnaire. The questionnaire took approximately 15 min to complete.

### Approach to Analysis

For the qualitative analysis of the responses to the open-ended questions that asked for a description of experiences with nature and shopping, we used a grounded, data-driven approach, chiefly guided by Strauss and Corbin (1990), i.e., our analysis was not organized according to *a priori* theoretical assumptions, but based on structures that emerged from the data. For the qualitative part of the analysis, in line with our objective to explore what ‘an experience that involves nature’ and ‘an experience that involves shopping’ meant to our respondents, this open-ended, exploratory approach seemed the most appropriate. In a first step, we explored the data to identify recurrent themes as well as striking arguments and patterns. These related to several different dimensions of the experiences described by our interviewees, including the places where the experience took place, the activities involved, the description of sensations and feelings, and social factors. In the nature-related experiences, encounters with non-human beings or particular features of nature were important part of the descriptions, whereas for the shopping experiences, the purpose of the activity (e.g., shopping for food, gifts, clothes, or groceries) played an important role in these accounts. Based on these themes, we then formed coding categories which constitute the backbone of the presentation of findings in the first two parts of the results section. These aim to provide an overview of the range of experiences that together constituted the two categories.

The questionnaire items were designed to elicit discrete ordered ratings on each of the semantic differential. Consequently, we use median values to describe them because they are more appropriate measures of central tendency for ordered data than mean values. We compare the scores on each individual semantic differential between experience categories using a Wilcoxon Signed-Ranks test, and in the regression model we focus on the semantic differential ‘pleasant–unpleasant,’ and model the response variable as ordinal.

**FIGURE 1:**
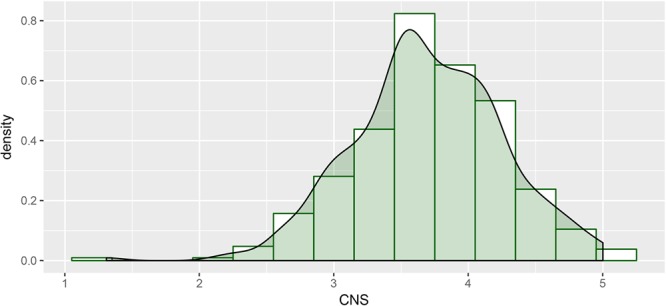
Histogram showing distribution of CNS scores, with transparent Gaussian kernel density estimate overlaid.

Prior to constructing the ordinal regression model (see below), in order to aid interpretation, we created a binned variable for connectedness to nature by dividing the distribution of CNS scores into terciles (with cutpoints at scores 3.38 and 3.9), ranging from ‘1’ representing low connectedness to ‘3’ representing high levels of connectedness. The raw data distribution (prior to binning) can be seen in **Figure 1**.

We then examined the relationship between people’s connectedness to nature and their ratings of how pleasant their experiences were for both categories (nature and shopping) via a cumulative logit model (Agresti, 2010) in which we accounted for the interaction between experience and CNS. Cumulative logit models are a form of ordinal response modeling which extends standard linear procedures in order to be able to account for the ordinal nature of the outcome variable.

In order to find a simplified model with the best possible fit, we tested the proportional odds assumption of equal slopes across the response categories. The assumption did not hold for our data; therefore, the final model specification is that of a partial proportional odds model where the assumption is relaxed for the categorical variable (i.e., factor) experience.

Additionally, the cumulative logit model takes into account that, by definition, the rating scale for the pleasantness variable is symmetric (that is, the end categories are perceived to be equally far from the central category). Cumulative logit models rely on the concept of thresholds on a continuous latent scale, which are cut-points defining the categories of the ordinal response variable. The threshold coefficients are parameters that allow us to calculate the points in the latent scale where responses might be predicted into a higher category and the corresponding slopes (like the intercept and slopes in a linear regression, except that each logit has its own). The relevance of these coefficients and one of the strengths of an ordinal model (versus a linear regression model) is that they allow us to calculate response category probabilities if required (Agresti, 2010; Christensen, 2015).

All statistical analyses were carried out in R (R Core Team, 2016), and the ordinal models were performed using the ordinal package (Christensen, 2015).

## Results

### Overview: Experiences That Involve Nature

All responses to our request to describe a recent “*experience which involved nature*” referred to direct, unmediated experiences – except for single statements, describing browsing through a nature book and composing a piece made out of birdsong, respectively – and nature outdoors – except for single contributions referring to the tending of indoor plants, and one on a visit to a zoo.

In the vast majority of cases, respondents described indeed – as framed by the questionnaire heading “Everyday experiences” – moments or activities that were part of their everyday life, such as dog walking, running or gardening. Only very few statements referred to experiences in an everyday environment that constituted extreme occasions: “*Being atop Bennachie* [a local hill, 528 m] *in a snow storm, the realization of how close to death I was truly opened my eyes*.”

Overall, this suggested that respondents felt that they had indeed something to say about ‘ordinary’ (as opposed to ‘spectacular’) nature experiences. Only very few respondents explicitly struggled with the question: “*This is difficult for me to think about because I feel that I do not experience nature very often. I guess to some extent watching my chameleon climbing around in its tank could be an experience of nature but like most other things its artificial and man made. Even when we think* [they] *are natural they are not really*.”

#### Sensory Dimensions of Experiences in Nature

Many responses referred to physical activities, such as running, hiking, going for a walk, or walking the dog. Others described experiences made in their own garden or allotment, but also local woodlands, beaches and urban parks were mentioned repeatedly. Strikingly, many statements mentioned sensations such as views, sounds, smells or the feeling of sun, grass or wind on one’s skin. Of particular relevance seemed to be sunshine and warm, dry weather. Seeing colors (such as a “wonderful fresh green”), trees, flowers, landscape (features) and animals were another very important element of our respondents’ sensory experiences. Most prominently, respondents described seeing and observing animals as their experience of nature – such sights could be fleeting (for example, while walking the dog) or targeted and over a longer period of time: “*I was watching some baby starlings being fed when I noticed one was sitting down and not moving. They all flew off together, including the seated one, only to return minutes later. The seated one again just flopped down and I realized it had either no legs or leg damage and therefore could not stand up. As the mother was feeding those which mobbed her and not the seated one, I am assuming that while he was in the nest, his chances were the same as the others, the problem arose once he left the nest. Felt quite sad after that*.”

Typically, such observations took place in the respondent’s garden: “*Watching a Thrush break a snail shell on a stone in my garden, it was completely absorbed with the task and totally unaware of me even though I came upon it in quite a noisy fashion.*”

And often, sensory experiences were seen to gain in meaning because of the respondent’s knowledge about its context: “*Driving near home and spotting a red kite flying over the field right by us. It was brilliant to see this magnificent bird for its own sake, and also because I know they have only recently been reintroduced to this area*.”

#### Emotional Dimensions

It seemed that respondents described experiences of interaction with animals in a particularly fond way: “*Successfully, after 5 years, teaching a blackbird to whistle the opening bars of Beethoven’s 5th Symphony, and also imitate me calling the cats, wolf-whistling. This happened a few years ago but to my mind is my greatest achievement!*”

This was particularly the case where this interaction involved the rescue of an animal: “*I found a small frog in my cellar yesterday which I was very pleased to be able to release into my garden*.”

In some cases, these feelings, as well as the sensory aspects of the experiences, triggered more abstract, situation-transcendent thoughts (as in the example of the snowstorm above) that tended to be positive and happiness-inducing: “*Being out for a walk with my dog and looking at the trees and wildlife that surrounded me. I find it stimulating to be out in the fresh air and relaxing when I’m in the fields and woods that surround my house. I appreciate the green vibrant landscape and it makes me feel connected with the earth and all that has to offer*.”

#### Social Dimensions

Many statements emphasized that being with friends or family was an essential part of the experience of nature, while others particularly valued solitude and the absence of other people (maybe apart from one’s companion). Several respondents described how they found solitude in an otherwise crowded (maybe peri-urban) environment by enjoying nature, for example, in the early morning, when no other people were around. For some parents, their children almost seemed to be proxies of their own nature experience: “*At the weekend I took my son and his friend to Drum castle so we could walk the dog. The boys spent a lot of time playing at the pond, clearing leaves and watching the water flow while I sat in the sunshine and the dog ran around exploring*.”

Only a few responses referred exclusively to inanimate nature, such as the sun. Interestingly, several respondents did not strictly distinguish between the ‘natural’ and human-made environment: “*My friend visited me in Aberdeen and we walked along the beach, appreciating the scenery. It was very enjoyable. The oil tankers were out and it was sunny so it was very picturesque!*”

Overall, it seemed that nature experiences were often described in very warm and fond terms. Even where experiences were negative, they were portrayed as sad (for example, seeing a dead animal) or as a challenge (such as getting wet on a hike, or feeling threatened by a snowstorm) rather than as outright unpleasant. In the majority of cases, gardens – as one’s own private, sheltered space – and parks, as well as other locations in the vicinity of the respondents were mentioned as the place where nature experiences took place. Such experiences were usually not intentional, but coincidental, occurring, for example, while going for a run or walk, and had very strong sensory components.

### Overview: Experiences That Involve Shopping

Responses to our request to describe an experience that involved shopping fell usually into one of three categories – the purchase of groceries in a supermarket, the purchase of groceries in a smaller shop, and the purchase of non-food items such as clothes. Of this last category several referred to experiences in other countries – however, being set in known types of environments such as malls, these were not necessarily portrayed as special or unique. Others described an online shopping experience, and a small number of participants chose to narrate an event which happened while they were shopping, but that in itself did not constitute an act of shopping, such as singing a love song to strangers in a supermarket, or observing a shoplifter. Interestingly, a large part of the responses focused on an unsuccessful search for an item that was wanted or needed.

#### Emotional Dimensions of Experiences Involving Shopping

Overall, a large part of the statements under this category did – as in the category of nature-related experiences – not include an evaluation (e.g., “*Searching for a dress to wear for a party*”; “*I went to buy shoes on Thursday after work and the shop was closed – it doesn’t open late on Thursdays*”). However, among those that included an assessment of the experience, many portrayed shopping as an admittedly necessary but frustrating and stressful “chore.” Many participants considered time spent on shopping as time wasted, and reducing the time needed for a shopping trip was regarded as an achievement. In line with this, shopping environments were typically described as busy and noisy places that triggered feelings of boredom, stress and time pressure. The phrase “I hate shopping” was repeatedly used, and terms such as “hurry,” “grab,” and “whizz” illustrated the prevalent perception of shopping taking up too much of one’s life time. Statements that described positive experiences often noted their surprise at a positive atmosphere which made the experience enjoyable: “…*and strangely it was very quiet and stress free. Even the staff were cheery*.”

Online shopping was regarded as a way out, providing for the necessary without the direct physical contact with shops and malls: “*Hate shopping, try to avoid it where possible but will shop on-line where I can*.”

For a few people, negative feelings while shopping were exacerbated by the more general, situation-transcendent thoughts that the activity triggered: “*Went to a superstore for the first time – it was surprising and depressing to think of myself as locked into a consumer society*.” Unlike for nature-related experiences where such more general thoughts were often positive, the more abstract considerations and reflections arising from shopping experiences thus tended to be critical of the economic system and society overall.

#### Social Dimensions

Where shopping was described as a positive experience, this was often due to the social character of the activity. A substantial part of the responses referred to the presence of others while shopping, and while many of these did not offer an explicit evaluation (“shopping with my flatmate, earlier today”), very few participants were outright negative about their shopping companions. Others described how knowing other customers and the staff of a shop made their shopping experience more pleasurable, and several participants mentioned how shopping helped them to bond with their teenage children – usually daughters: “*Shopping with my daughter on Monday. She is rarely at home so shopping together was lovely. For once, she accepted my suggestions and I hers*.”

Overall, experiences that involved shopping were thus described as more stressful and oppressing than experiences that involved nature, which were generally characterized as restorative. This might have been partly due to the perception of shopping as a duty, necessity or chore, whereas being in nature – at least among our study participants – was usually by choice. In both cases, social aspects played an important role, and while crowded environments were often referred to as stress-inducing, being with friends and family usually enhanced an experience and made it pleasant. This seemed especially important for the relationship between teenage daughter and parent in the case of shopping, whereas comments on experiences in nature usually referred to younger children.

### Comparing Ratings of Experiences

**FIGURE 2:**
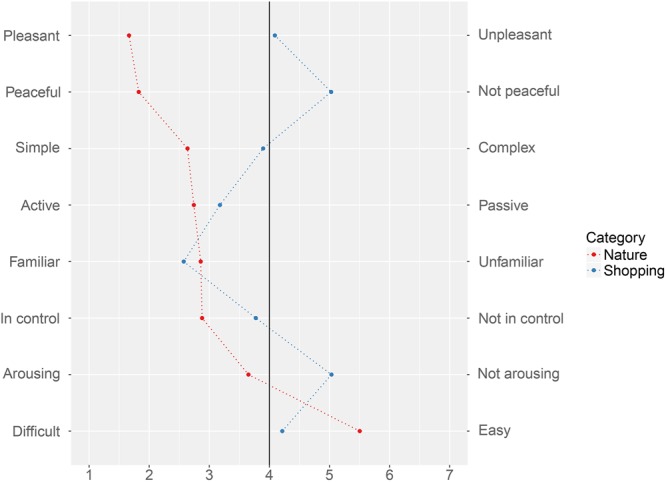
Semantic differential profile for the two experience categories. Points represent mean scores (used for illustrative purposes – see text for median), and dotted lines are included for each category to improve legibility.

Overall, experiences with nature tended to be perceived significantly differently from shopping experiences (**Figure 2**). When filling in the semantic differential scales immediately after providing their experience description, a Wilcoxon Signed-Ranks test indicated that on average participants reported their recollected ‘nature’ experiences as being significantly more pleasant (*Mdn* = 1) than their recollected ‘shopping’ experiences (*Mdn* = 4), *Z* = -12.77, *p* < 0.01, *r* = -0.68. Participants also reported ‘nature experiences’ as being significantly more peaceful (*Mdn* = 1) than shopping experiences (*Mdn* = 5), *Z* = -14.76, *p* < 0.01, *r* = -0.78. On average, participants found their recalled ‘nature’ experiences to be more arousing (*Mdn* = 3) than their ‘shopping’ experiences (*Mdn* = 6), *Z* = -9.34, *p* < 0.01, *r* = -0.49, and more active (*Mdn* = 2) than their shopping experiences (*Mdn* = 3), *Z* = -3.3, *p* < 0.01, *r* = -0.17. Participants also experienced nature experiences as simpler (*Mdn* = 2) than shopping experiences (*Mdn* = 4), *Z* = -8.56, *p* < 0.01, *r* = -0.45, and ‘easier’ *Mdn* = 6) than shopping experiences (*Mdn* = 4), *Z* = -8.47, *p* < 0.01, *r* = -0.45.

### The Relationship between Perceptions of Experiences and Connectedness to Nature

There was a statistically significant association (at 5% significance level) between CNS and the rating of pleasantness of the recalled nature experience (*r*_s_ = -0.23, *p* < 0.01), with people with higher scores on CNS rating nature experiences as more pleasant.

As noted in the approach to analysis section, in order to aid interpretation of the ordinal model, we created a binned variable for connectedness to nature by dividing the distribution into terciles. The cumulative logit model (**Table 1**) showed that there was a statistically significant interaction between connectedness to nature and experience category (shopping versus nature), both when comparing medium to low, and high to low levels of CNS. Essentially this means that people who scored high on connectedness to nature were likely to feel more pleasure when experiencing nature, and less pleasure when shopping. Similarly, people with low scores on connectedness to nature were likely to report higher levels of pleasure from shopping experiences, and lower pleasure with nature experiences. However, in absolute terms, nature experiences were for all three groups more pleasurable than shopping experiences.

**Table 1:** Results of partial proportional odds model with symmetric thresholds.

	Estimate	Standard error	*z*-value	*p*
**Coefficients:**				
CNS2 (medium)	0.71	0.27	2.64	0.008
CNS3 (high)	1.16	0.28	4.07	<0.001
**Interactions:**				
CNS2 (medium) × category (shopping)	–0.91	0.36	–2.52	0.012
CNS3 (high) × category (shopping)	–1.91	0.37	–5.17	<0.001
**Threshold coefficients:**				
*Intercepts*				
Central 1	–2.08	0.26	–8.11	
Central 2	–1.59	0.22	–7.39	
Spacing 1	0.38	0.09	4.42	
Spacing 2	1.42	0.14	9.89	
*Slopes (nature as reference)*				
Central 1	1.47	0.31	4.69	
Central 2	1.68	0.28	6.03	
Spacing 1	0.22	0.10	2.15	
Spacing 2	–0.02	0.17	–0.13	

*Reference category for connectedness is low and for category of experience is nature. Dependent variable is rated pleasantness.*

**FIGURE 3:**
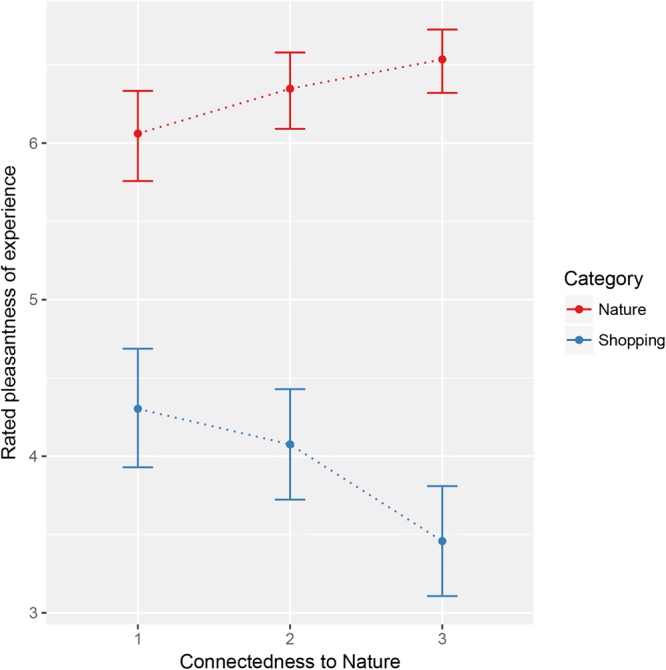
Mean plot of rated pleasantness across CNS levels for the two experience categories. Error bars represent 95% bootstrapped confidence intervals based on 10,000 resamples.

This difference is clearly discernible in **Figure 3** which also shows evidence of the interaction between experience and CNS.

## Discussion

We set out to explore how certain categories of experience compare to each other from the perspective of an individual, with a specific focus on two categories – namely ‘nature’ and ‘shopping.’ Our findings suggest that experiences of local, accessible nature that can be used on a daily basis are extremely valuable and meaningful to people, and can provide deep emotional experiences. Whilst we found that our comparison category of everyday experience, shopping, tended to be generally less positive in emotional terms, there was a notable level of variability in the responses. We found that some of this variability could be accounted for by connectedness to nature. Moreover, we found an interaction between category of experience (in this case nature or shopping) and the relationship between connectedness to nature and the rated pleasantness of the recalled experience. The finding that higher levels of nature connectedness are associated with greater levels of pleasantness in recalled nature-related experiences is what we would expect from the literature on connectedness, as this is implied by the link to psychological wellbeing in much of the writings on this topic (see e.g., Mayer and Frantz, 2004; Zhang et al., 2014). The inverse relationship that we find here with the category shopping is not, however, something that is obviously predicted from the literature on connectedness, but our finding suggests that people high in nature connectedness may have a lower likelihood of finding shopping experiences pleasurable, as the category of experience is seemingly opposed in terms of (e.g., environmental) orientation, being focused on the domain of material consumption. Indeed, research on materialism suggests that people holding materialistic values tend to shop more (Goldberg et al., 2003). Moreover, if one considers this from the perspective of psychological values set out by Schwartz (1992), materialism would be expected to be negatively correlated with the value of universalism (Stern et al., 1999), and positively correlated with extrinsic values (see e.g., Brown et al., 2016). A fruitful avenue for future research would be to try and better understand how the structure of psychological values is associated with the types of responses people have to different categories of everyday experience. Indeed, in order to adequately account for the individual variation in responses to the category shopping, it would be worthwhile to develop a measure of ‘connectedness to shopping,’ or possibly something at a higher level of generality such as ‘affinity to an urban lifestyle.’

Although the world we live in is increasingly defined by its urbanized character, and lower levels of direct contact between people and nature (Hartig et al., 2014), this study found that people’s memories of ‘experiences which involved nature’ were associated with a number of positive emotions – far more so than for ‘experiences which involved shopping.’ However, care needs to be taken not to over-interpret this finding. By comparing at the level of the category, we inevitably avoided the methodological straightjacket associated with fixing the experience itself, but in doing so, we allowed a large source of variation to be introduced in terms of explaining the responses. ‘Nature’ it seems is not an immutable category, and should not be treated as one.

Similarly, in the eyes of our study participants, certain types of shopping were more pleasurable than others, and the analysis presented here cannot adequately capture this important source of variability. In our data, experiences such as shopping for groceries in a crowded supermarket tended to be less likely to be positive than, for example, shopping for a gift for a loved one. Indeed, studies such as Jones (1999) and Arnold et al. (2005) have highlighted the factors that appear to distinguish pleasant from unpleasant shopping experiences. In themselves though, such observations do not adequately explain the interaction we found. People who scored high on connectedness to nature found, on average, their reported shopping experience less pleasurable compared to people with low scores, although of course we need to be careful not to over-interpret this, as participants were only requested to provide a single exemplar for each category of experience.

Regarding the descriptions given by participants of everyday experiences, an important distinction needs to be made between what might be thought of as being spectacular or extraordinary experiences and more mundane, everyday experiences. However, some of the examples given by our respondents powerfully illustrate that special experiences can also be had in mundane and well-known environments. Focusing on the everyday is not only academically interesting, but also important from an applied perspective: Whilst it might not be possible to design interventions to give people the opportunity to experience ‘spectacular nature,’ or even spectacular experiences in everyday environments, it seems useful to think of ways to increase the opportunities to experience everyday nature more frequently, such that the occurrence of memorable and positive experiences becomes more likely.

Accounting for differences in participants responses to the two category prompts from a psychological perspective is difficult, partly because there is still a lack of an encompassing psychological theory to draw on that adequately accounts for category-level descriptions and specific exemplars at the same time (Murphy, 2016). Even though we find a notable difference in the average ratings of pleasantness associated with those experiences described in response to the category prompts ‘Shopping’ and ‘Nature,’ the magnitude of the difference is less important than the variability of the ratings. As noted earlier, some of this variability can be attributed to the experience itself, but there are also individual differences that account for important parts of the variation in responses – such as the interaction between nature connectedness and the category of experience described in our study, but there are many others that would be interesting to consider, including aspects of personality and value orientations for example. Moreover, important social and demographic aspects need to also be accounted for. It may be for example that a person with children is more likely to respond to certain experiences in a different manner to those without children, as our qualitative findings suggest, where experiences in nature shared with young children were reported on, whereas shopping might be more likely to be actively shared with teenage sons or daughters.

As well as not accounting for such aspects, there are of course several limitations to the exploratory study presented within this paper. Firstly, the fact that we asked people to focus on a single event, so we cannot know how representative this single event is of their portfolio of experiences in response to either category prompt. This is something that should ideally be addressed in future research designed to explore some of these issues in more depth. The other limitation, as described in the methods section, was that the order of presentation was always the same – so responses to the category prompt ‘nature’ were always made before responses to the category prompt ‘shopping.’ We therefore cannot rule out the existence of an order effect in our study. To some extent, the fact that the first questions focused on nature experiences (rather than shopping) might also have led to a self-selection of nature-interested respondents, so any follow-up study should randomize the category order to enable any such effects to be ruled out.

In relation to the issue of question order, it is of course possible that the recollection of nature-related experiences in the first part of the questionnaire acted as a prime for later responses in the questionnaire on the connectedness to nature scale. Mayer et al. (2009) found that recent exposure to nature resulted in increases in measures of connectedness to nature, so there is a possibility that the task of recalling a nature experience had a priming effect, which would require future research to clarify. In addition, the exploratory nature of this study, and the fact that our sampling strategy focused on maximizing variability in response, meant that the sample was not representative of the population. In order to extrapolate from findings like this – for example to inform policy recommendations – it would be necessary to carry out future studies using a sampling approach designed around the principles of representativeness.

One area of research that may benefit from considering the findings presented here is the study of pro-environmental behavior. Studies have reported findings showing a positive association between connectedness to nature and pro-environmental behavior (Mayer and Frantz, 2004; Barbaro and Pickett, 2016). Other studies (e.g., Corral-Verdugo et al., 2010) have shown that some shopping-related beliefs (particularly austerity beliefs concerning buying only strictly necessary items) and pro-environmental behaviors (especially waste-reduction behaviors) are strongly related. Joining up the investigation of nature connectedness with the study of everyday consumption behaviors (i.e., shopping) is potentially a useful starting point in addressing the important question of how attitudes toward nature and nature conservation relate to pro-environmental attitudes and behaviors.

## Conclusion

This study has explored the variation of responses to two common categories of everyday experience – namely nature and shopping. Experiences involving nature are often drawn on as a given category in environmental psychology (often in opposition to the built environment). Here we go beyond this and provide insights into the variability of remembered experiences associated with both ‘nature’ and ‘shopping.’ Although nature experiences were generally found to be more pleasant and to bring to mind more positive memories compared to shopping experiences, the results were far from clear-cut.

More research is needed here, but this study has demonstrated that the two categories studied here, nature and shopping, provide an interesting point of comparison when examining people’s responses to different categories of everyday experience.

## Ethics Statement

The wider project of which this study was part (RES-355-25-0012) underwent ethical review, and the study was carried out in accordance with the recommendations of the ethics committee of the project coordinator with written informed consent from all subjects. All subjects gave written informed consent in accordance with the Declaration of Helsinki. At the time that the research was carried out, The James Hutton Institute did not have its own research ethics committee, but the protocol for the core questionnaire used across the wider project was approved by the research ethics committee of the project coordinator, and this study did not deviate from this protocol.

## Author Contributions

TC was the principal investigator for this study. He conceived and designed the study and led the writing of the paper. AF contributed to the early discussions of the study design, analyzed the qualitative data, and contributed to the preparation of the manuscript. AL-A carried out important parts of the statistical analysis, and contributed to the preparation of the manuscript.

## Conflict of Interest Statement

The authors declare that the research was conducted in the absence of any commercial or financial relationships that could be construed as a potential conflict of interest.
